# Successful treatment of recurrent small cell carcinoma of urinary bladder with pembrolizumab

**DOI:** 10.1002/iju5.12208

**Published:** 2020-08-05

**Authors:** Tomoya Hatayama, Tetsutaro Hayashi, Shinji Matsuzaki, Hiroshi Masumoto, Hiroyuki Yanai, Hamidreza Abdi, Jun Teishima, Yasuhisa Hasegawa

**Affiliations:** ^1^ Department of Urology National Hospital Organization Fukuyama Medical Center Hiroshima Japan; ^2^ Department of Urology Hiroshima University Hospital Hiroshima Japan; ^3^ Department of Pathology Okayama University Hospital Okayama Japan; ^4^ Department of Surgery Division of Urology University of Ottawa Ottawa Ontario Canada

**Keywords:** bladder cancer, immune checkpoint inhibitor, immunotherapy, small cell carcinoma of urinary bladder, urothelial carcinoma

## Abstract

**Introduction:**

Small cell carcinoma of urinary bladder is rare and has an aggressive malignant behavior and poor prognosis. Advanced bladder cancers are treated with immune checkpoint inhibitors, however, its efficacy for small cell carcinoma of urinary bladder is unclear.

**Case presentation:**

A 54‐year‐old female, diagnosed with clinical stage T2N0M0 small cell carcinoma of urinary bladder, underwent radical cystectomy after three cycles of etoposide‐cisplatin neoadjuvant chemotherapy. Despite the fact that pathological examination revealed no residual carcinoma in bladder in her cystectomy specimen, local recurrence of a 60‐mm mass detected in the follow‐up investigation 7.5 months later. This was completely treated by pembrolizumab without any adverse effects. Immunohistochemical staining revealed that the tumor had no programmed death ligand 1 expression but it showed CD8‐positive T‐lymphocyte infiltration into the tumor.

**Conclusion:**

Immune checkpoint inhibitors might have curative potentials for treatment of small cell carcinoma of urinary bladder.

**Abbreviations & Acronyms**
CT: computed tomography
ICI: immune checkpoint inhibitor
LRC: laparoscopic radical cystectomy
MRI: magnetic resonance imaging
NAC: neoadjuvant chemotherapy
NSE: neuron specific enolase
ORR: overall response rate
PD‐1: programmed death 1
PD‐L1: programmed death ligand 1
SCCB: small cell carcinoma of urinary bladder
SCLC: small cell lung cancer
TURBT: transurethral resection of bladder tumor
UC: urothelial carcinoma

**Keynote message**

SCCB has an aggressive malignant behavior and has been usually treated with chemotherapy extrapolated from SCLC. We reported a successfully treated case which showed ICI may be considered as a novel therapeutic option for the treatment of SCCB.

## Introduction

SCCB is a rare disease with an aggressive malignant behavior and poor prognosis.^1^, ^2^, ^3^ Because of limited number of SCCB cases, standard treatment for SCCB has not been established yet and most of them have been treated with chemotherapy extrapolated from treatment of SCLC with or without surgery or radiotherapy.^4^ However, the efficacy of treatment is unclear and further studies are needed to find the best treatment option.^2^

Pembrolizumab is an ICI medication which targets the PD‐1 receptor and has been approved for treatment of patients with advanced stage UC and SCLC. However, the evidence for its efficiency in patients with SCCB is lacking. In this report, we describe a case of SCCB who had local recurrence after LRC with NAC which completely responded to pembrolizumab.

## Case presentation

**Fig. 1:**
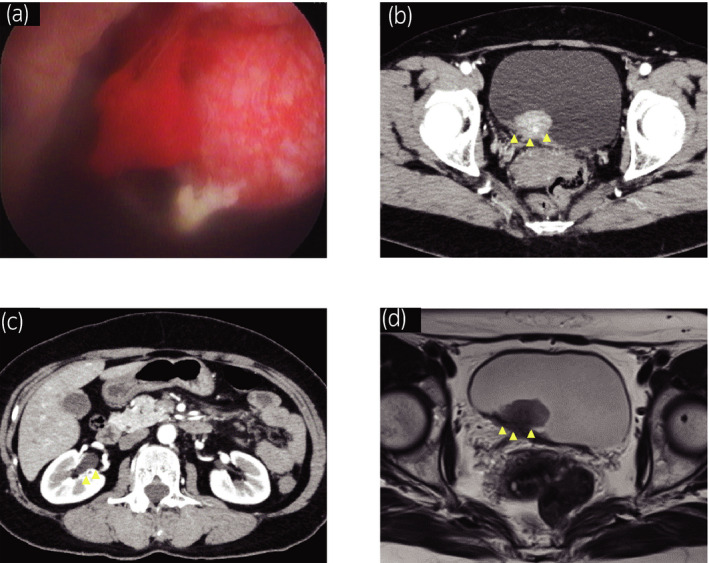
Clinical and radiological findings before TURBT. (a) Cystoscopy showed a sessile non‐papillary bladder tumor adjacent to the right ureteric orifice. (b) Abdominal CT with contrast showed a 20‐mm bladder tumor at the right posterior bladder wall and (c) Grade 1 right hydronephrosis. (d) Abdominal MRI showed a 20‐mm bladder tumor suspected for invasion to the muscular layer at the right posterior bladder wall on T2 weighted image.

A 54‐year‐old female with gross hematuria and dysuria was referred to our hospital with the suspicion of bladder cancer. Urine analysis confirmed hematuria with pyuria and urine cytology showed class 5. Ultrasonography and cystoscopy revealed the presence of a non‐papillary bladder tumor adjacent to the right ureteric orifice (Fig. 1a **)**. CT scans and MRI showed a 20‐mm bladder tumor at the right posterior bladder wall suspected for muscle invasion and grade 1 right hydronephrosis (Fig. 1b–d). There was no evidence of distant or lymph node metastases.

**Fig. 2:**
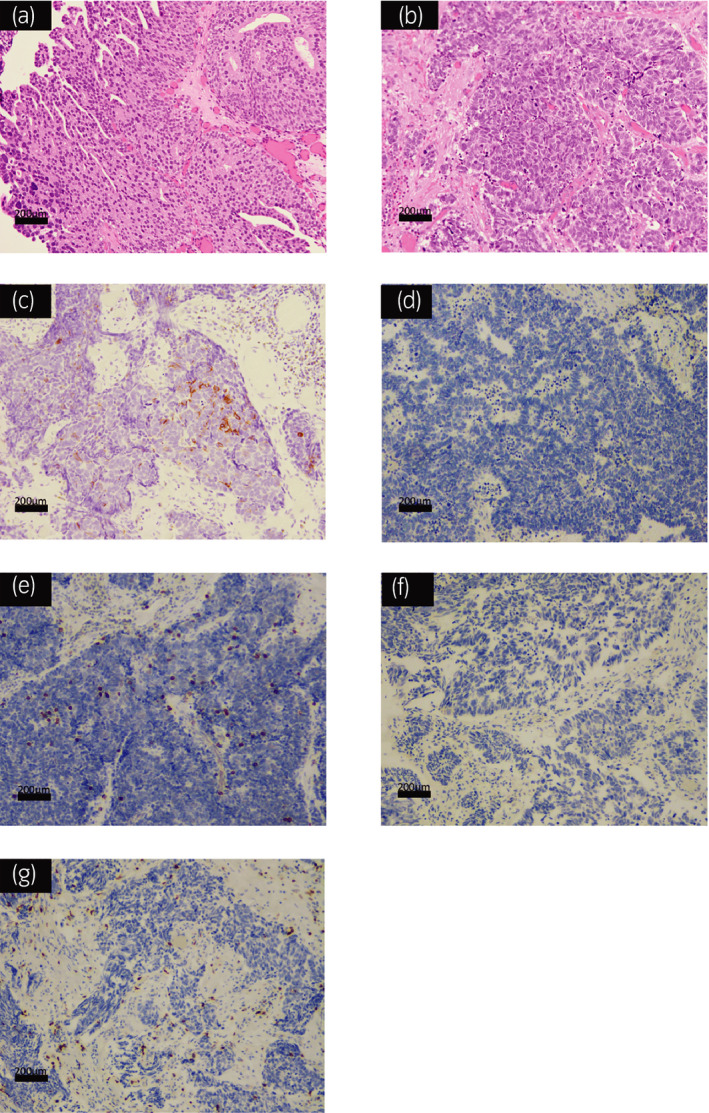
Pathological findings of hematoxylin and eosin staining and immunohistochemical special staining. (a) Hematoxylin and eosin staining of the bladder tumor showed both the urothelial cell carcinoma component and (b) small cell carcinoma component. (c) Immunohistochemical staining of the small cell carcinoma component showed that CD56 is partially positive, (d) PD‐L1 is negative. (e) Tumor infiltration of CD8^+^ T lymphocytes into tumor was observed. (f) PD‐L1 is also negative and (g) tumor infiltration of CD8^+^ T lymphocytes into tumor was observed in UC components.

TURBT was performed and pathological examination showed evidence of muscle invasion with UC accompanied by small cell carcinoma (Fig. 2a,b). Immunohistochemical staining showed that CD56 is partially positive (Fig. 2c) while Chromogranin A and Synaptophysin were negative on the specimen. TURBT, serum NSE level was within the normal level (6.1 ng/ml).

Given her clinical staging (T2N0M0), we planned for three cycles of etoposide‐cisplatin NAC‐based treatment of SCLC. After NAC, CT scans showed decreasing size of bladder tumor and once again we did not detect any distant and lymph node metastases. Then, LRC with extended pelvic lymph node dissection and ileal conduit diversion was performed. Pathological finding revealed no residual carcinoma in the bladder with no lymph node metastases, pT0N0M0.

**Fig. 3:**
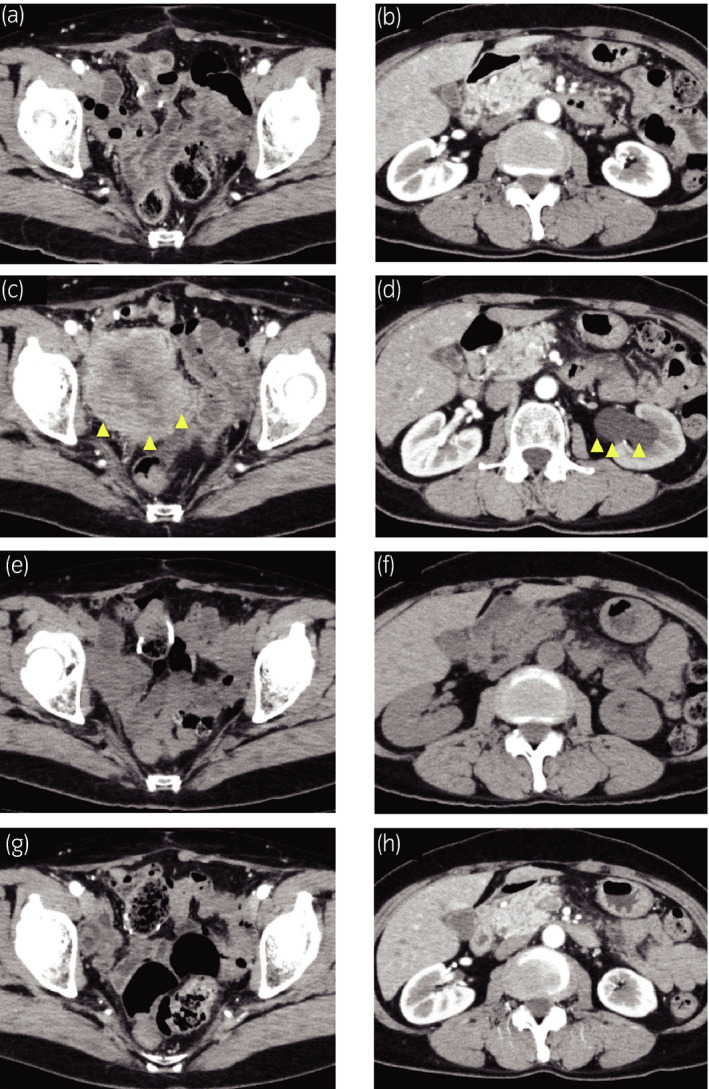
Radiological findings after LRC. (a) Abdominal CT showed no recurrent tumor 4 months after LRC. (b) No evidence of left hydronephrosis 4 months after LRC. (c) Abdominal CT with contrast showed a 60‐mm mass at the pelvis and (d) left hydronephrosis 7.5 months after LRC. (e) Abdominal CT showed disappearance of recurrent tumor at the pelvis and (f) left hydronephrosis after two cycles of pembrolizumab therapy. (g, h) Abdominal CT with contrast showed no tumor recurrence after five cycles of pembrolizumab therapy.

In the follow‐up imaging 4 months after LRC, there was no evidence of disease recurrence (Fig. 3a,b). However, 7.5 months after LRC, serum NSE level increased up to 45.7 ng/ml. CT scans showed a 60‐mm mass at the pelvis with possible invasion to the left distal ureter (Fig. 3c,d). This local recurrence of SCCB was discussed with the patient, she decided to proceed with pembrolizumab. After receiving two cycles of pembrolizumab therapy, CT scans demonstrated disappearance of recurrent tumor and left hydronephrosis (Fig. 3e,f). After completing five cycles of pembrolizumab therapy, local recurrent tumor and left hydronephrosis remained undetectable on CT scans (Fig. 3g,h). We evaluated efficacy of pembrolizumab therapy as complete response by the Response Evaluation Criteria in Solid Tumors 1.1^5^ and serum NSE level decreased to the normal level (10.2 ng/mL). Twelve cycles of pembrolizumab therapy are ongoing and she has no adverse events.

## Discussion

SCCB accounts for less than 1% of all the bladder cancers and predominantly occurs in men (3:1 male‐to‐female ratio) at the 6th decade of their lives. In most cases, they initially present with gross hematuria and have muscle invasive stage T2 or higher at the time of diagnosis.^6^, ^7^ The standard treatment protocol is lacking and the prognosis of SCCB is poor, and 5‐year survival rate of all comers are somewhere between 16 and 25%.^8^, ^9^

According to the 2019 National Comprehensive Cancer Network guidelines in bladder cancer, initial chemotherapy followed by radiotherapy or cystectomy is recommended for patients with SCCB with localized disease regardless of stage.^10^ A recent systematic review also showed that NAC and radical cystectomy could have some benefits for patients with early‐stage SCCB.^11^ Although systematic chemotherapy with the agents used for SCLC are recommended for patients with SCCB with distant or lymph node metastases in the National Comprehensive Cancer Network guidelines, the 5‐year survival rates for patients with stage IV were 10.5% despite appropriate recommended therapy in a large series.^8^

Two studies looked at the ORR of ICIs for patients with stage IV UC with neuronal subtypes including SCCB (Table 1).^12^, ^13^ One of them reported relatively high ORR to Atezolizumab.^12^ The other case report showed reasonable response to pembrolizumab in a patient with chemotherapy refractory SCCB.^14^ The complete response to immunotherapy in our case after relapse is consistent with previously described results in those studies.

**Table 1:** Overall response rate of ICIs for patients with stage 4 neuronal subtype UC including SCCB

Author	Patients	Treatment	Number of patients	Response	
				ORR (%)	Best response
Kim *et al*.^12^	Patients with platinum‐refactory or cisplatin‐ineligible neuronal subtype UC	Atezolizumab	11	72%	Complete response 2
					Partial response 6
					Not able to assess 3
Miller *et al*.^13^	Patients who received anti‐PD‐1 or PD‐L1 for UC with neuroendcrine feature	Atezolizumab	6	25%	complete response + partial response 2
		Durvalumab	1		No response 6
		Nivolumab	1		Not able to assess 1
		Pembrolizumab	1		

Some previous studies looking into pathologic features of responders to immunotherapy suggested that PD‐L1 expression, tumor infiltration with CD8^+^ T lymphocytes and tumor mutational burden are predictive biomarkers in ICI therapy.^15^, ^16^ PD‐L1 expression and tumor mutational burden have been also suggested as predictive biomarkers of response to immunotherapy for patients with SCLC.^17^ It is however interesting to note that immunohistochemical staining of neuroendocrine bladder tumor including SCCB has shown less expression of PD‐L1 and density of CD8^+^ T lymphocyte infiltration.^18^ In our case, we also performed immunohistochemical staining of specimens resected by TURBT. We found that PD‐L1 expression was negative in both SCCB and UC components; however, tumor infiltration with CD8^+^ T lymphocytes was observed in both components (Fig. 2d–g). Tumor infiltration with CD8^+^ T lymphocytes may be the reason that pembrolizumab had responded in our case.

Using our case and mentioned small series, we can conclude that cancer microenvironment needs more future attention and deserves performing prospective trials to test the ability of immunotherapy in SCCB. Recently, a phase II study (NCT03430895) planned to evaluate ICIs therapy in patients advanced non‐pure UC including SCCB and will hopefully answer more questions regarding this novel therapeutic option.^19^

## Conclusion

We presented a case of SCCB with local recurrence after NAC followed by LRC and responded totally to pembrolizumab. Treatment protocol for SCCB and this successfully treated case suggest that immunotherapy can be considered as a possible effective treatment option for patients with SCCB.

## Ethics

We obtained written informed consent from the patient.

## Conflict of interest

The authors declare no conflict of interest.

## References

[1] Siegel RL , Miller KD , Jemal A . Cancer statistics, 2020. CA Cancer J. Clin. 2020; 70: 7–30. 31912902 10.3322/caac.21590

[2] Niu Q , Lu Y , Xu S *et al* Clinicopathological characteristics and survival outcomes of bladder neuroendocrine carcinomas: a population‐based study. Cancer Manag. Res. 2018; 10: 4479–89. 30349380 10.2147/CMAR.S175286 PMC6190820

[3] Humphrey PA , Moch H , Cubilla AL , Ulbright TM , Reuter VE . The 2016 WHO Classification of tumours of the urinary system and male genital organs‐part B: prostate and bladder tumours. Eur. Urol. 2016; 70: 106–19. 26996659 10.1016/j.eururo.2016.02.028

[4] Kouba EJ , Cheng L . Understanding the genetic landscape of small cell carcinoma of the urinary bladder and implications for diagnosis, prognosis, and treatment: a review. JAMA Oncol. 2017; 3: 1570–8. 28334324 10.1001/jamaoncol.2016.7013

[5] Eisenhauer EA , Therasse P , Bogaerts J *et al* New response evaluation criteria in solid tumours: revised RECIST guideline (version 1.1). Eur. J. Cancer 2009; 45: 228–47. 19097774 10.1016/j.ejca.2008.10.026

[6] Erdem GU , Özdemir NY , Demirci NS , Şahin S , Bozkaya Y , Zengin N . Small cell carcinoma of the urinary bladder: changing trends in the current literature. Curr. Med. Res. Opin. 2016; 32: 1013–21. 26889739 10.1185/03007995.2016.1155982

[7] Schreiber D , Rineer J , Weiss J *et al* Characterization and outcomes of small cell carcinoma of the bladder using the surveillance, epidemiology, and end results database. Am. J. Clin. Oncol. 2013; 36: 126–31. 22391430 10.1097/COC.0b013e3182438c71

[8] Choong NW , Quevedo JF , Kaur JS . Small cell carcinoma of the urinary bladder. The Mayo Clinic experience. Cancer 2005; 103: 1172–8. 15700264 10.1002/cncr.20903

[9] Cheng L , Pan CX , Yang XJ *et al* Small cell carcinoma of the urinary bladder: a clinicopathologic analysis of 64 patients. Cancer 2004; 101: 957–62. 15329903 10.1002/cncr.20456

[10] National Comprehensive Cancer Network . Bladder Cancer (Version 4.2019). 2019 [Cited 10 Aug 2019.] Available from URL: https://www2.tri‐kobe.org/nccn/guideline/urological/english/bladder.pdf

[11] Veskimäe E , Espinos EL , Bruins HM *et al* What is the prognostic and clinical importance of urothelial and nonurothelial histological variants of bladder cancer in predicting oncological outcomes in patients with muscle‐invasive and metastatic bladder cancer? A European Association of Urology muscle invasive and metastatic bladder cancer guidelines panel systematic review. Eur. Urol. Oncol. 2019; 2: 625–42. 31601522 10.1016/j.euo.2019.09.003

[12] Kim J , Kwiatkowski D , McConkey DJ *et al* The cancer genome atlas expression subtypes stratify response to checkpoint inhibition in advanced urothelial cancer and identify a subset of patients with high survival probability. Eur. Urol. 2019; 75: 961–4. 30851984 10.1016/j.eururo.2019.02.017

[13] Miller NJ , Khaki AR , Diamantopoulos LN *et al* Histologic subtypes and response to PD‐1/PD‐L1 blockade in advanced urothelial cancer: a retrospective study. J. Urol. 2020; 204: 63–70. 31971495 10.1097/JU.0000000000000761 PMC7289665

[14] Wilde L , Ali SM , Solomides CC , Ross JS , Trabulsi E , Hoffman‐Censits J . Response to pembrolizumab in a patient with chemotherapy refractory bladder cancer with small cell variant histology: a case report and review of the literature. Clin. Genitourin. Cancer 2017; 15: e521–e524. 28143711 10.1016/j.clgc.2016.12.012

[15] Tumeh PC , Harview CL , Yearley JH *et al* PD‐1 blockade induces responses by inhibiting adaptive immune resistance. Nature 2014; 515: 568–71. 25428505 10.1038/nature13954 PMC4246418

[16] Topalian SL , Hodi FS , Brahmer JR *et al* Safety, activity, and immune correlates of anti‐PD‐1 antibody in cancer. N. Engl. J. Med. 2012; 366: 2443–54. 22658127 10.1056/NEJMoa1200690 PMC3544539

[17] Gelsomino F , Lamberti G , Parisi C *et al* The evolving landscape of immunotherapy in small‐cell lung cancer: A focus on predictive biomarkers. Cancer Treat. Rev. 2019; 79: 101887. 31491661 10.1016/j.ctrv.2019.08.003

[18] Mandelkow T , Blessin NC , Lueerss E *et al* Immune exclusion is frequent in small‐cell carcinoma of the bladder. Dis. Markers 2019; 2019: 2532518. 31191745 10.1155/2019/2532518 PMC6525886

[19] ClinicalTrials.gov . Evaluating Immune Therapy, Durvalumab (MEDI4736) With Tremelimumab for Metastatic, Non‐transitional Cell Carcinoma of the Urinary Tract. [Cited 20 Feb 2020.] Available from URL: https://clinicaltrials.gov/ct2/show/NCT03430895.

